# Effective norm emergence in cell systems under limited communication

**DOI:** 10.1186/s12859-018-2097-2

**Published:** 2018-04-11

**Authors:** Xiaotian Hao, Jianye Hao, Li Wang, Hanxu Hou

**Affiliations:** 1 School of Computer Science and Software, Tianjin University, Peiyang Park Campus: No.135 Yaguan Road, Haihe Education Park, Tianjin, 300350 China; 2 School of Electrical Engineering and Intelligentization, Dongguan University of Technology, No. 1, university road, songshan lake district, dongguan, 221116 China

**Keywords:** Cell system, Cooperative multi-agent system, Reinforcement learning, Social norms, Limited communication

## Abstract

**Background:**

The cooperation of cells in biological systems is similar to that of agents in cooperative multi-agent systems. Research findings in multi-agent systems literature can provide valuable inspirations to biological research. The well-coordinated states in cell systems can be viewed as desirable social norms in cooperative multi-agent systems. One important research question is how a norm can rapidly emerge with limited communication resources.

**Results:**

In this work, we propose a learning approach which can trade off the agents’ performance of coordinating on a consistent norm and the communication cost involved. During the learning process, the agents can dynamically adjust their coordination set according to their own observations and pick out the most crucial agents to coordinate with. In this way, our method significantly reduces the coordination dependence among agents.

**Conclusion:**

The experiment results show that our method can efficiently facilitate the social norm emergence among agents, and also scale well to large-scale populations.

## Background

**Fig. 1:**
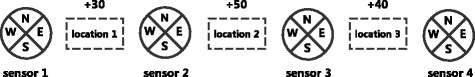
Sensor network with 4 sensors

All living systems live in dynamical environments. The biological system behaviors [1–9] result from the interactions among millions of cells and their environments. For example, The human immune system is designed to protect us from infection by many different kinds of organisms, including bacteria, fungi and parasites. The immune process is the interaction and cooperation of different immune cells. Different cells have different functions, and the cooperation of the different cells makes up life. Similarly, a cooperative multi-agent system (MAS) [10, 11] is composed of a set of autonomous agents that interact with each other within their communication capacity to reach a common goal or to optimize the global performance. For example, in the sensor network shown in Fig. 1, to reach the accuracy, two sensors are needed to observe the same place. If location 1, location 2 and location 3 always have targets with resulting reward +30, +50, +40 respectively, then by using the independent policy sensor 2 and sensor 3 prefer to observe the location 2 for a higher reward +50. However, the optimal policy is sensor 1 and sensor 2 always observing location 1 and sensor 3 and sensor 4 always observing location 3 which results in the highest global reward +70.

In the research of the cooperative MAS, social norms play an important role in regulating agents’ behaviors to ensure coordination among the agents. For example, in our life, we should drive on the left (or right) according to the traffic rules. When it comes to biological systems, this corresponds to coordinating on the well-coordinated states for better survival. In biology, different cells are designed for different functions and cells should coordinate their functions to ensure that the overall biological system functions correctly.

Many researches have investigated biological systems which are composed of cells and environments via modeling and simulation [1, 12]. If we regard cells in biological system as agents in multi-agent system, the well-coordinated states among cells can be viewed as social norms in multi-agent systems. Thus, investigating how social norms can emerge efficiently among the agents in multi-agent systems would provide valuable insights for better understanding how cells can interact to achieve well-coordinated states. One commonly adopted description of a norm is that a norm serves as a consistent equilibrium that all agents follow during interactions where multiple equivalent equilibriums may exist. Until now, significant efforts have been devoted to studying norm emergence problem [13–20]. However, most of the existing approaches require significant communications and intensive computations.

Considering the fact that the communications between the cells are limited in biological systems (by sending electrical or chemical signals), we develop a learning approach based on the individually learning methods and the DCOP algorithm under limited communication bandwidth to facilitate the norm emergence in agent societies. In many practical applications, although the agents may interact with many others over time to make a better decision, they usually only need to coordinate with very few agents which strongly affect their performance. Based on previous research [21, 22], we first define a criteria to measure the importance of different subgroup of neighbors by estimating the maximum potential utility each subgroup can bring. Based on this, each agent can estimate the utility loss due to the lack of coordination with any subgroup of agents. Furthermore, each agent dynamically selects the best subset of neighbors to coordinate with for minimizing the utility loss. At last, each agent trades off learning performance and communication cost by limiting the maximum of the miscoordination cost. Experiments results indicate that (1) with the limited communication bandwidth and in different networks (e.g., regular network, random network, small-world network, scale-free network) our method can efficiently facilitate the emergence of norms compared with the existing approaches. (2) Our method allows agents to trade off the norm emergence performance and the communication cost by adjusting the parameters. (3) Compared with the previous methods, our method can significantly reduce the communication cost among agents and result in efficient and robust norm emergence.

The remainder of this paper is organized as follows. “Methods” section first discusses the basic domain knowledge, and then formally gives the definition of the single state coordination problem and the symbolic representation, and at last presents the architecture and the details of our method. “Results and discussion” section presents experimental evaluation results. Finally, we conclude in “Conclusion” section.

## Methods

### Game theory and Nash equilibrium

#### Game theory

Game theory is a mathematical theory concerned with the optimum choice of strategy in situations involving a conflict or cooperation of interest (Also called theory of games). To be fully defined, a game must specify the following elements.

- players, the players of the game.
- actions, the actions available to each player at each decision point.
- payoffs, the feedback of making a decision and taking the selected action.
- strategies, also called policy, is a high level plan to achieve the goal under conditions of uncertainty.

#### Normal form games

**Fig. 2:**
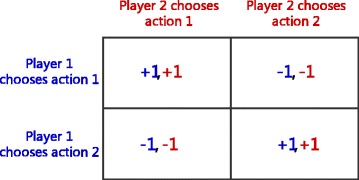
Payoff matrix of a 2-player, 2-action normal form game
- *A*_*i*_, a finite of actions for each player *i*.
- *A*, *A*=*A*_1_×…×*A*_*n*_ is the set of joint actions, where × is the Cartesian product operator.
- *R*_*i*_, *A*_1_×…×*A*_*n*_→*R*, the reward received by agent *i* with a join action $\vec a \in A$.
- *π*_*i*_, *A*_*i*_→[ 0,1], the probability of player *i* to select each action in *A*_*i*_.
- pure strategy, *π*(*a*_*k*_)=1 for action *a*_*k*_,and for other actions *π*(*a*_*j*,*j*≠*k*_)=0.
- mixed strategy, the probability of selecting an action is under some distribution. And the pure strategy is a special case of the mixed strategy.

The normal (or strategic form) game is usually represented by a matrix which shows the players, strategies, and payoffs (see Fig. 2 for an example). More generally it can be represented by any function that associates a payoff for each player with every possible combination of actions. Usually, the normal form game can be represented as a tuple (*n*,*A*_1,…,*n*_,*R*_1,…,*n*_),

- 1,…,*n*, n players of the game.

#### Nash equilibrium

Use a two-player normal form game with pure strategy to describe the definition.

- Best Response:
  when player 1 selects an action *a*_1_, the best response of player 2 is that player 2 select an action which maximizes its reward, that means *a*_2_=argmax_*a*__2_∈*A*_2_*R*_2_.
- Nash Equilibrium:
  If each player has chosen a strategy and no player can benefit by changing strategies while the other players keep theirs unchanged, that means the chosen action for each player is the best response to the other player’s choice, then the current set of strategy choices and the corresponding payoffs constitutes a Nash equilibrium.

### Reinforcement learning

#### Markov decision process

A basic Markov Decision Process (MDP) can be represented as a tuple (*S*,*A*,*T*,*R*),

- *S*, a finite set of states representing the state space.
- *A*, a finite set of actions for the agent.
- *T*, a state transition probability function, *T*:*S*×*A*×*S*→[ 0,1], which specifies the probability of transition from state *s*∈*S* to *s*^′^∈*S* when action *a*∈*A* is taken by the agent. Hence, *T*(*s*,*a*,*s*^′^)=*Pr*(*s*^′^|*s*,*a*).
- *R*, a reward function $R:S \times A \times S \rightarrow \mathbb {R}$, the immediate reward for being in state *s*∈*S* and taking the action *a*∈*A* and then transfer to state *s*^′^∈*S*.

When the state, action, transition function and the reward function are all known, we can use some searching methods (e.g., Monte Carlo Tree Search) to solve the problem. And this is one of the classes of reinforcement learning, saying model-based methods. And the other one is model-free, which means the model is unknown.

#### Introduction of reinforcement learning

In simple terms, reinforcement learning (RL) is a class of methods that the agent continuously interacts with the environment and according to the feedback reward, dynamically adjusts its policy to maximize the expectation of the long-term feedback reward. Explore the environment through trial and error, the methods will gradually improve its performance and finally converge to an optimal policy. Trail and error and the delayed reward is important characteristics of the RL. RL methods always include the 4 basic elements: (1) agent: subject of learning and the object interacting with the environment. (2) environment: the environment that the agents reside in (static and dynamic). (3) action space: the actions available for an agent at certain states (discrete or continuous). (4) feedback reward: a method to measure the utility of an action at certain states.

#### Q-learning

Q-Learning is an important milestone of RL study which is a kind of model-free methods. It’s the alias of the *TD*(0). The core equation of Q-Learning can be described as:

1 $$ {\begin{aligned} Q(s_{t},a_{t})&=Q\left(s_{t},a_{t}\right)\\ & \quad +\alpha \left[r_{t}+\gamma \max_{a_{t+1}}Q\left(s_{t+1},a_{t+1}\right)-Q\left(s_{t},a_{t}\right)\right] \end{aligned}}  $$

where *α*∈ [0,1] is the learning rate, *r*_*t*_ is the immediate reward of doing *a*_*t*_ at state *s*_*t*_, *γ*∈[ 0,1] is the discount factor, which is usually set to 1 for a finite horizon. *Q*(*s*_*t*_,*a*_*t*_) is the state-action value function, which represents the expectation of the long-term accumulated feedback reward when in state *s*_*t*_ and selects action *a*_*t*_. An typical procedure of Q-Learning is described as Algorithm 1.



### Topology of networks

#### Regular network

**Fig. 3:**
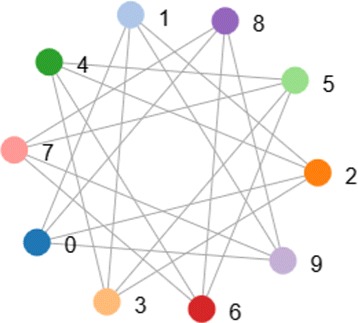
Regular network

Regular network is built upon ring network, in which each node (n nodes in total) connect with the nearest m nodes. And when *m*=*n*−1, it’s a fully-connected network. See Fig. 3 for an example.

#### Random network

**Fig. 4:**
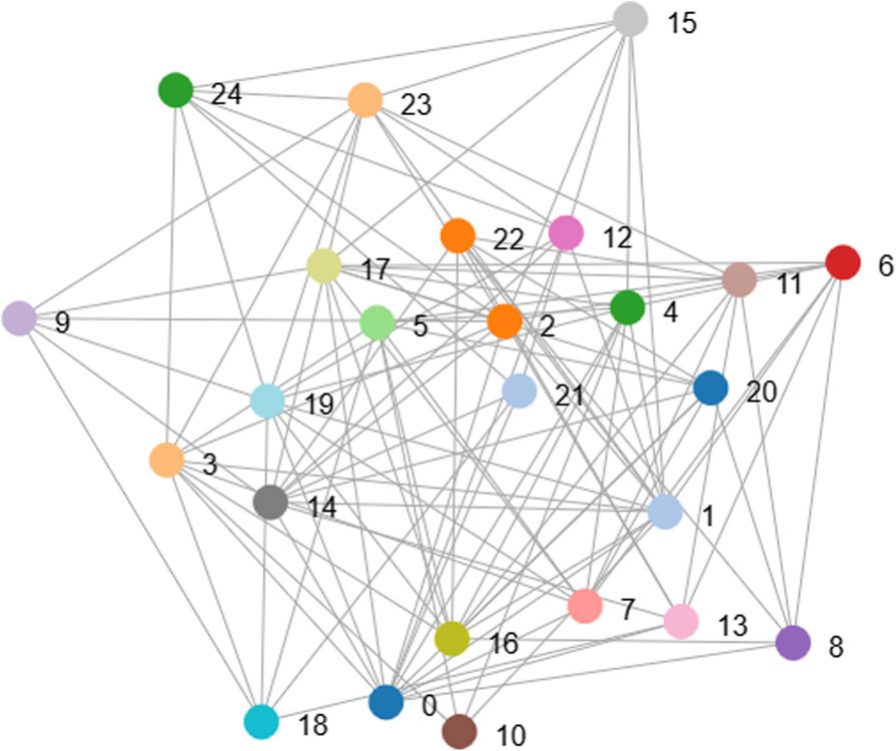
Random network

Random graphs may be described simply by a probability distribution, or by a random process which generates them. A typical model is the ER-model in which each edge has a fixed probability of being present or absent, independently of the other edges. See Fig. 4 for an example.

#### Small world network

**Fig. 5:**
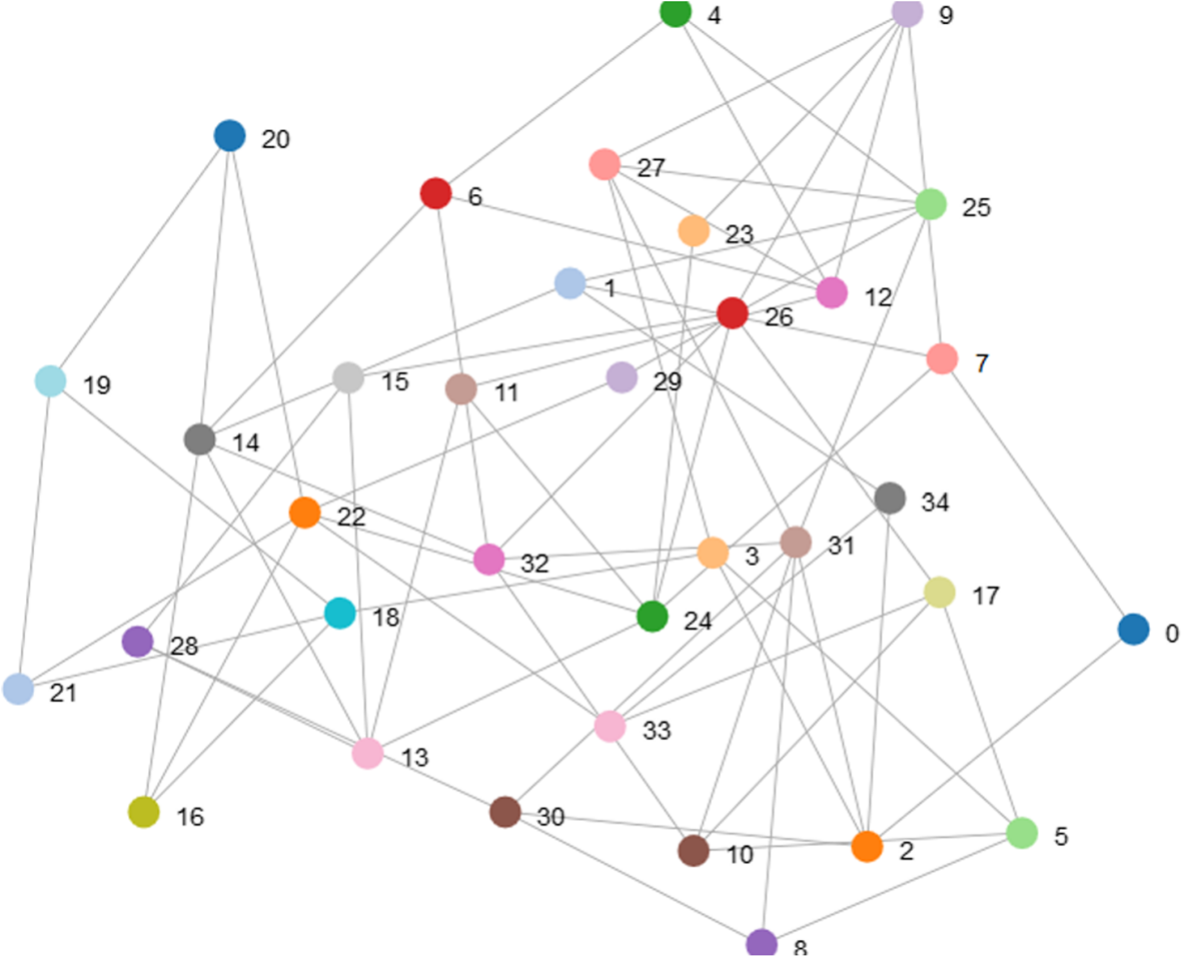
Small world network

Small-world network is proposed to describe the interpersonal relationship in which each person is a node, and the relationship (e.g., familiar or not) between two persons is an edge. A certain category of small-world was developed by Duncan Watts and Steven Strogatz. See Fig. 5 for an example.

#### Scale free network

**Fig. 6:**
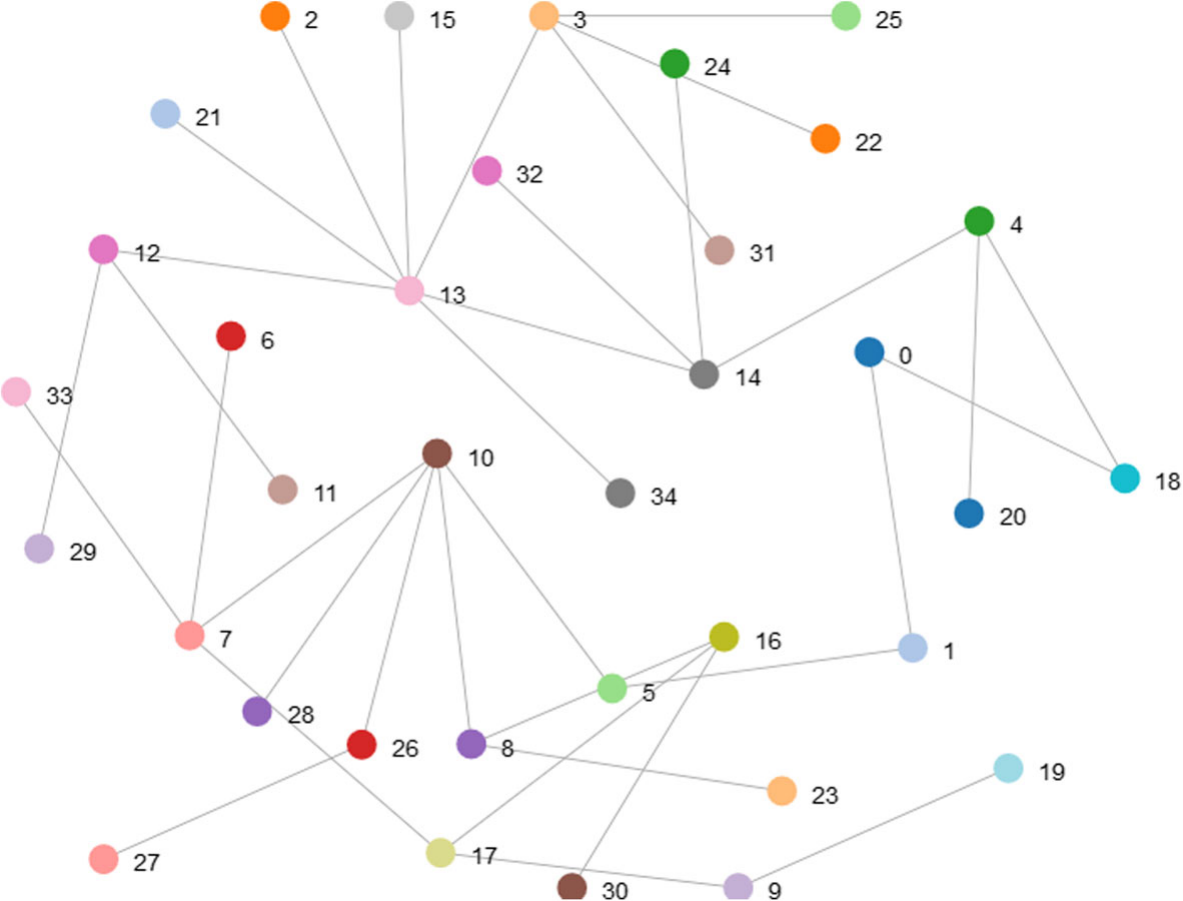
Scale free network

The nodes in scale-free network do not connected randomly. Only a few of nodes serve as the center of the graph which have higher degree and the others connect with fewer nodes. See Fig. 6 for an example.

### Coordination problem

In cooperative multi-agent systems, agents share common interests. The agent will make its choice according to the neighbors’ actions. Each agent in the environment makes a choice and selects an action *a*_*i*_ at each time step, then the join action is $\vec a=(a_{1},\ldots,a_{n})$, and afterwards, the whole receives a join reward $R(\vec a)$. The target of the coordination problem is to find the best $\vec {a^{*}}$ which maximizes the total reward $R(\vec a)\ \left (\vec {a^{*}}={\text {argmax}}_{\vec a}R(\vec a)\right)$. For the sake of exposition, we define a cooperative multi-agent problem with only one state for each agent. Each of the two adjacent agents play a two-agent n-action normal form game. In a two-player, two-action, general-sum normal-form game, the payoff for each player can be specified by a matrix as show in Fig. 2. The agents have the same action space. When the adjacent agent *i*,*j* select the same action, they will both receive a reward of *r*(*a*_*i*_,*a*_*j*_)=+1, otherwise *r*(*a*_*i*_,*a*_*j*_)=−1. We assume that agent *i* can observe each neighbor’s action selection during the interaction and so that can get some statistical information of each neighbor. The symbols used in the following sections are described bellow.

- *n*, number of agents.
- *A*_*i*_, the action space of each agent *i*.
- *S*_*i*_, the state space of each agent *i*, each agent only have one state here, that means no state transition.
- *r*_*i*_, the immediate reward of agent *i*.
- *π*_*i*_, the policy of agent *i*, *π*_*i*_→*a*_*i*_.
- $\vec A$, $\vec A=A_{1} \times... \times A_{n}$, the joint action space of all agents.
- $\vec S$, the joint state space of all agents.
- *Q*_*i*_(*s*,*a*), the local expectation of the discounted reward for agent *i* selecting action *a* in state *s*.
- $Q(\vec s,\vec a)$, the global expected reward of selecting joint action $\vec A$ in joint state $\vec S$.
- *τ*(*i*), all neighbors of agent *i*.
- *CS*(*i*), the coordination set of agent *i*, and agent *i* should coordinate its action selection with the agents in *CS*(*i*), *CS*(*i*)⊆*τ*(*i*).
- *NC*(*i*), the neighbors of agent *i* that are not in *CS*(*i*), *NC*(*i*)=*τ*(*i*)∖*CS*(*i*).
- *CG*, coordination graph which is composed of the *CS* of all agents.

### Coordinated learning with controlled interaction

#### Coordination graph

**Fig. 7:**
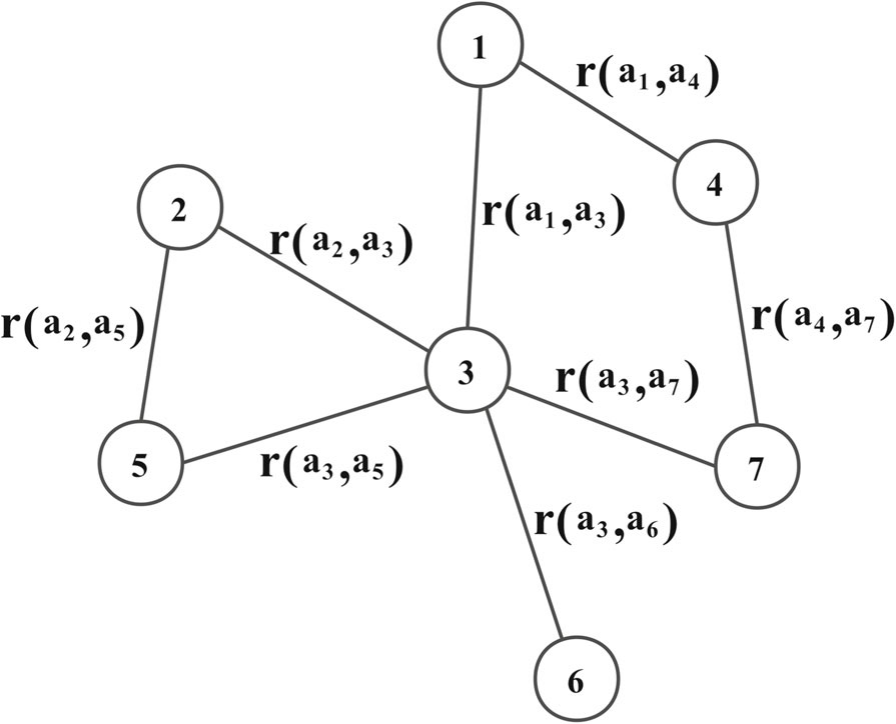
Coordination graph

To solve the coordination problem, one straightforward way is to loop through all the possible $\vec A$ and select $\vec A$ which maximizes the total reward. However this is practically intractable, due to the huge search space exponential to the number of agents (which is |*A*_1_×…×*A*_*n*_|) and the agents might not have access to the needed information (e.g., all other agents’ actions and rewards). Luckily, in practice, each agent’s choice only depends on a small set of relevant agents. The coordination graphs (CGs) described by Guestrin et al. [23] is a typical solution for this policy dependency problem. In a coordination graph *G*=(*V*,*E*) as shown in Fig. 7, each node represents an agent, and each agent *i*’s reward only depend on the adjacent agents. Each edge (*i*, *j*)∈*E* represents that the relevant agents *i*, *j* have to coordinate their actions, and the related value *r*(*a*_*i*_,*a*_*j*_) is the reward agent *i*, *j* will receive when selecting action *a*_*i*_,*a*_*j*_ respectively. The total reward $R(\vec {a})$ is the sum of the individual reward *r*(*a*_*i*_,*a*_*j*_), as shown in Eq. (2).

2 $$  R(\vec{a})=\sum_{(i,j)\in E}^{n}r(a_{i},a_{j})  $$

#### Cooperative Q-learning

**Fig. 8:**
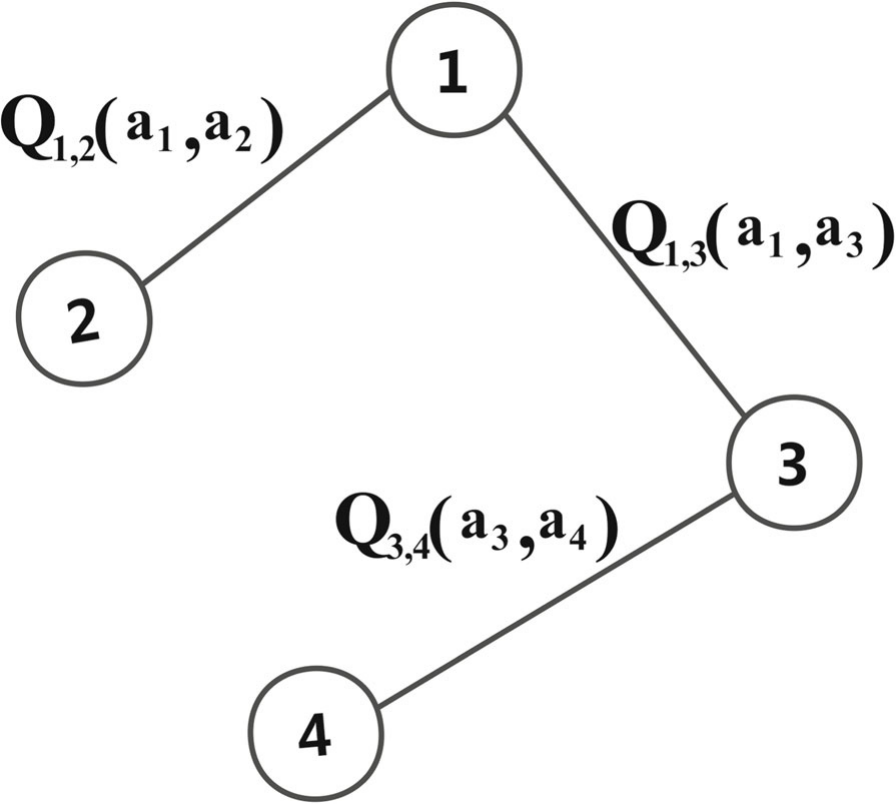
Coordination graph with Q

We use Q-learning to estimate the expectation of the long-term feedback reward of the adjacent agents *i*,*j* choosing action *a*_*i*_,*a*_*j*_, the bounded reward value in the edge of the coordination graph ((*i*,*j*)∈*E*) is represented by *Q*(*a*_*i*_,*a*_*j*_). An example of the modified coordination graph is shown in Fig. 8. Our purpose is to find a policy that maximizes the overall expected utility $Q(\vec a)\left (\pi ={\text {argmax}}_{\vec a \in A} Q(\vec a)\right)$. The global Q-learning update rule is shown in Eq. (3).

3 $$ {\begin{aligned} Q(\vec s_{t},\vec a_{t})&=Q\left(\vec s_{t},\vec a_{t}\right)\\ & \quad +\alpha \left[r_{t}+ \gamma \max_{\vec a_{t+1}}Q\left(\vec s_{t+1},\vec a_{t+1}\right)-Q\left(\vec s_{t},\vec a_{t}\right)\right] \end{aligned}}  $$

Although the global join learning approach leads to an optimal policy, it is practically intractable. In practice, it’s possible to approximate the global utility $Q(\vec a)$ by the sum of the individual utility. Then, $Q(\vec a)$ can be represented as:

4 $$  Q(\vec s_{t},\vec a_{t})=\sum_{(i,j)\in E} Q_{ij}\left(s_{i,j}^{t},a_{i}^{t},a_{j}^{t}\right)  $$

The global Q-Leaning update rule shown in Eq. (3) can be rewritten as:

5 $$ \begin{aligned} \sum_{(i,j)\in E} Q_{ij}\left(s_{i,j}^{t},a_{i}^{t},a_{j}^{t}\right) &=(1-\alpha)\sum_{(i,j)\in E} Q_{ij}\left(s_{i,j}^{t},a_{i}^{t},a_{j}^{t}\right)\\ & \quad + \alpha \left[\sum_{(i,j)\in E} r_{a_{i},a_{j}}^{t}+\gamma \max{\vec a_{t+1}}Q\left(\vec s_{t+1},\vec a_{t+1}\right)\right] \end{aligned}  $$

where $r_{a_{i},a_{j}}^{t}$ is the reward the adjacent agents *i*,*j* receive when selecting the actions $a_{i}^{t},a_{j}^{t}$ respectively. Note that the $\max {\vec a_{t+1}}Q(\vec s_{t+1},\vec a_{t+1})$ cannot be directly decomposed into the sum of the local discounted future rewards, for it depends on the global joint action $\vec A$ which maximizes the global utility $Q\left (\vec s_{t+1},\vec a_{t+1}\right)$. We should find the optimal joint action $\vec {a}^{*}$ where $\vec {a}^{*} = (\text {argmax})_{\vec a}Q\left (s_{t+1},\vec a\right)$. For $\vec {a}^{*}$ is a vector and can be represented by $\left (a_{1}^{*},\ldots,a_{n}^{*}\right)$, $\max _{\vec a_{t+1}}Q\left (s_{t+1},\vec a_{t+1}\right) = Q\left (s_{t+1},\vec {a}^{*}\right)=\sum _{(i,j)\in E} Q_{ij}\left (s_{i,j}^{t+1},a_{i}^{*},a_{j}^{*}\right)$. So, for each pair of agents, we have

6 $$ {\begin{aligned} Q_{ij}\left(s_{i,j}^{t},a_{i}^{t},a_{j}^{t}\right)&=(1-\alpha)Q_{ij}\left(s_{i,j}^{t},a_{i}^{t},a_{j}^{t}\right)\\ & \quad + \alpha \left[r_{a_{i},a_{j}}^{t}+\gamma Q_{ij}\left(s_{i,j}^{t+1},a_{i}^{*},a_{j}^{*}\right)\right] \end{aligned}}  $$

What’s remaining unknown in Eq. (6) is the optimal action $a_{i}^{*}$ for each agent *i*. Since enumerate all the combinations of the $\vec a^{*}$ is intractable, we use the message-passing DCOP algorithm to find the optimal action $a_{i}^{*}$ for each agent *i* in next section.

#### Coordinated action selection

**Fig. 9:**
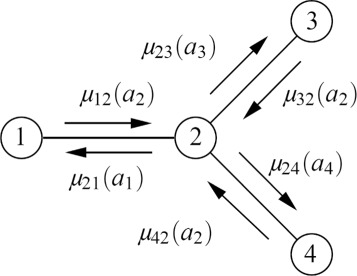
Message passing over a 4-agent graph

We use the Max-Plus algorithm proposed by J. R. Kok and N. Vlassis. [21] to find $a_{i}^{*}$ for each agent *i*. To compute the optimal $\vec a^{*}$ for the whole, each agent sends a message to each of its neighbors. The definition of the message from agent *i* to agent *j* is defined as follows.

7 $$ \mu_{ij}(a_{j})=\max_{a_{i}}\left\{Q_{ij}(a_{i},a_{j}) + \sum_{k \in CS(i)\backslash j} \mu_{ki}(a_{i}) \right\} + c_{ij}  $$

where *CS*(*i*)∖*j* is the coordinated neighbors of agent *i* except *j*, *μ*_*ki*_(*a*_*i*_) is the messages from agent *i*’s neighbors (except j) to *i* and the parameter *c*_*i*,*j*_ is a standardization item to prevent the value of the message being overflow. Notice that for a given message *μ*_*ij*_(*a*_*j*_), the value only depends on the target agent *j*’s action *a*_*j*_. Given an action *a*_*j*_, the sender *i* can make a best response to maximize the value of *μ*_*ij*_(*a*_*j*_). Each agent *i* in the CG will continuously send an message *μ*_*ij*_(*a*_*j*_) to each of its neighbor *j* at every decision point until the value of the message converges to a stable value or the available time slots are used up or the agent receives some termination signal. When the messages over the whole network all become stable, each message will contain the *Q*_*ij*_(*a*_*i*_,*a*_*j*_) value bounded in every edges (*i*,*j*)∈*E*. Therefore, maximizing the sum of the current messages received from neighbors is to maximize the global $Q(\vec a)$ for each agent. Figure 9 gives an example of the message passing over a 4-agent coordination graph. So for each agent *i*, the best action $a_{i}^{*}$ to maximize the global utility is

8 $$ a_{i}^{*}=(\text{argmax})_{a_{i}}\sum_{k \in CS (i)} \mu_{ki}(a_{i})  $$

Above all, the algorithm for each agent *i* to get the optimal action $a_{i}^{*}$ is described in Algorithm 2. For more details on max-plus, refer to J. R. Kok and N. Vlassis’s paper [21].



#### Coordination set selection: random

For large problems, the messages passed in the network are directly proportional to the number of edges of the CG but the communication is limited. To reduce the communication times and frequency, we need to eliminate some non-critical edges of the CG without significantly affecting the system performance. In this subsection, we define 2 different methods to minimize the communication cost.

In this subsection, we use some random methods to reduce the communication frequency.

- Random agents: For each agent *i*, during the learning process, only *δ* percent of its neighbors *τ*(*i*) are selected as the *CS*(*i*).

In addition to the Random methods, we add some decay here.

- Random agents with decay: We first initialize an *δ*=*δ*_0_. During the learning process, we randomly select *δ* percent of the neighbors *τ*(*i*) as the *CS*(*i*) for each agent *i* at each decision point. And then we decrease the *δ* with some small decay (e.g., *δ*=*δ*−0.01). With time going by, the *δ* will be smaller and smaller until to the minimum value specified (e.g., 0).

#### Coordination set selection: loss rate

To reduce the communication without significantly affecting the system performance, we need to find out the difference of communicating with an agent or not. For this purpose, we divide the neighbors *τ*(*i*) of each agent *i* into two groups: *CS*(*i*) and *NC*(*i*) as mentioned before. Each agent *i* only has to communicate with the agents in *CS*(*i*) to coordinate their actions.

For agents in *CS*(*i*), we assume that they have coordinated their actions well with agent *i*, and each of them will try their best to maximize the total reward of the group. And for agents in *NC*(*i*), each agent *i* will calculate the expectation of the reward when *a*_*i*_ is selected. $Q_{i}(a_{i})=\sum _{k\in NC(i)} \sum _{a_{k} \in A_{k}}P_{k}(a_{k}|a_{i})Q_{ik}(a_{i},a_{k})$, where *P*_*k*_(*a*_*k*_|*a*_*i*_) is the probability of neighbor *k* selecting action *a*_*k*_ when agent *i* selects *a*_*i*_. For a selected *CS*(*i*), the potential expected utility of selecting action *a*_*i*_*PV*(*a*_*i*_,*CS*(*i*)) is divided into two parts: agents in *CS*(*i*) and agents in *NC*(*i*).

9 $$ \begin{aligned} PV_{i}(a_{i},CS(i))& =\sum_{j\in CS(i)}\max_{a_{j}}Q_{ij}(a_{i},a_{j}) \\ & \quad +\sum_{k\in NC(i)} \sum_{a_{k} \in A_{k}}P_{k}(a_{k}|a_{i})Q_{ik}(a_{i},a_{k}) \end{aligned}  $$

Obviously, if *CS*_1_(*i*)⊆*CS*_2_(*i*)⊆*τ*(*i*), then for an action *a*_*i*_, *PV*_*i*_(*a*_*i*_,*CS*_1_(*i*))≤*PV*_*i*_(*a*_*i*_,*CS*_2_(*i*)).

Based on the potential expected utility, we define the potential loss in lack of coordination with *NC*(*i*) (*PL*_*i*_(*NC*(*i*)) for each agent *i*. It’s the difference of the potential expected utility when agent *i* coordinates with all of its neighbors *τ*(*i*) from that of agent *i* when it only coordinates with *CS*(*i*).

10 $$ \begin{aligned} PL_{i}(NC(i))&=\max_{a_{i},a_{i} \in A_{i}}PV_{i}(a_{i},\tau(i)) \\ &\quad - \max_{a_{i},a_{i} \in A_{i}}PV_{i}(a_{i},CS(i)) \end{aligned}  $$

Easily, we can find that (1) if *NC*_1_(*i*)⊆*NC*_2_(*i*)⊆*τ*(*i*), then *PL*_*i*_(*NC*_1_(*i*))≤*PL*_*i*_(*NC*_2_(*i*)). (2) *PL*_*i*_(*∅*)=0. (3) for each *NC*(*i*)⊆*τ*(*i*),0≤*PL*_*i*_(*NC*(*i*))≤*PL*_*i*_(*τ*(*i*)).

Above all, each agent *i* will select the best coordination set *CS*(*i*) according to the *PL*(*τ*(*i*)∖*CS*(*i*)) to minimize the loss of utility. The algorithm is described in Algorithm 3. *δ* is the predefined loss rate. When *δ*=0, each agent *i* will coordinate with all neighbors and when *δ*=1, each agent *i* will not coordinate with any agent at all.



#### Learning processes with emergent coordination

Combining cooperative Q-learning, coordinated action selection, and the coordination set selection, the cooperative learning process is described in Algorithm 4.



## Results and discussion

In this section, we evaluate the performance of our algorithm on a large single-state problem. Firstly, we give the common settings of the large single-state problem. Then, we compare the norm emergence performance of our algorithm with some existing approaches. At last, we explore the effect of some important parameters and the performance of different coordination set selection methods proposed in “Coordination set selection: random” and “Coordination set selection: loss rate” sections.

### Large scale single-state problems

There is only one state for each agent, and the reward function is defined in “Coordination problem” section (See Fig. 2 for an example). The goal of the agents is to learn and select a joint action which maximizes the global reward. In the following subsections, without additional explanation, we consider 100 agents playing a 10-action coordination game in which 10 norms exist. And the agents distribute in a small-world network. The average connection degree of the graph is set to 6.

### Norm Emergence Performance

In this subsection, we compare the norm emergence performance of our methods with two of the existing approaches. For it’s difficult for the other two approaches to reach the convergence, the number of agents used here is 50 and the action number used is 2. The other parameter settings are shown in Table 1.

- Independent Learners (IL): Each agent *i* uses the independent Q-learning and adjusts its policy only depend on its own action and reward. The Q-function is updated according to Eq. (11).
  11 $$ {\begin{aligned} Q_{i}(s,a_{i})&=Q_{i}(s,a_{i})\\ & \quad +\alpha\left[r\left(s,a,s^{\prime}\right)+ \gamma \max_{a_{i}^{\prime}}Q_{i}\left(s^{\prime}, a_{i}^{\prime}\right)-Q_{i}\left(s^{\prime},a_{i}^{\prime}\right)\right] \end{aligned}}  $$

**Table 1:** Parameter settings for “Norm Emergence Performance” section

Parameter name	Value
Agent number	50
Action number	2
Init explore rate	1.0
Delta explore rate	0.004 (IL:0.04)
Init learning rate	1.0
Delta learning rate	0.0005
Min learning rate	0.6
Message differ(max-plus)	0.00001
Message sent deadline(max-plus)	5
- Distributed Value Functions (DVF): Each agent *i* records a local Q-function based on its own action and reward, and updates it incorporating with the neighbors’ Q-function following equation 12. *f*(*i*,*j*) is the contribution rate of agent *j* to agent *i*, and here is 1/|*τ*(*i*)|. For the stateless problem, we make an adjustment that each agent select its action considering the neighbors’ Q-function, that is $a_{i}^{*}={\text {argmax}}_{a\in A_{i}}\sum _{j\in \{(i) \cup \tau (i)\}}f(i,j) \max _{a_{j}^{\prime }}Q_{j}\left (s^{\prime }, a_{j}^{\prime }\right)$.
  12 $$ {\begin{aligned} Q_{i}(s,a_{i})&=Q_{i}(s,a_{i})\\ &\quad +\alpha \!\left[\!r\left(s,a,s^{\prime}\right)\,+\, \gamma \!\sum_{j\in \{(i) \cup \tau(i)\}}f(i,j) \max_{a_{j}^{\prime}}Q_{j}\left(s^{\prime}, a_{j}^{\prime}\right)\,-\,Q_{i}\left(s^{\prime},a_{i}^{\prime}\right)\!\right] \end{aligned}}  $$

**Fig. 10:**
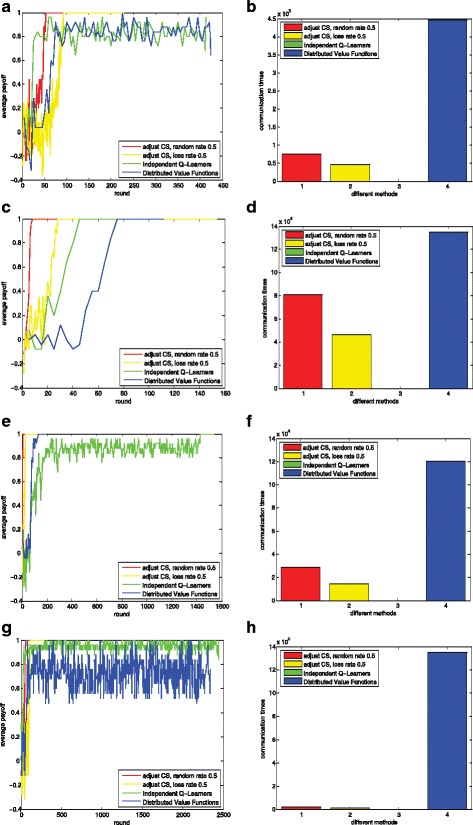
Norm emergence performance under different network topologies. Figure 10**a** Learning process (regular network); Fig. 10**b** Communication times (regular network); Fig. 10**c** Learning process (random network); Fig. 10**d** Communication times (random network); Fig.10**e** Learning process (small-world network); Fig. 10**f** Communication times (small-world network); Fig. 10**g** Learning process (scale-free network); Fig. 10**h** Communication times (scale-free network)

The norm emergence performance and the corresponding communication times are shown in Fig. 10. The learning processes are shown in the left parts and the corresponding message passing times over all agents are shown in the right parts. Our methods show better learning performance over all networks. We find that only in random network, all the methods lead to quick norm emergency as shown in Fig. 10c. In regular network, small-world network and scale-free network, only our methods converge to a global optimal in a few steps as shown in Fig. 10a, e and g. The communication cost of our method is much smaller than that of DVF (For IL, communication is not needed).

### Influence of key parameters

In this section, we investigate the influence of some key parameters to the performance of norm emergence and message passing times. The parameters of the compared algorithm are the same other than the comparison one.

#### The influence of random parameter *δ*

**Fig. 11:**
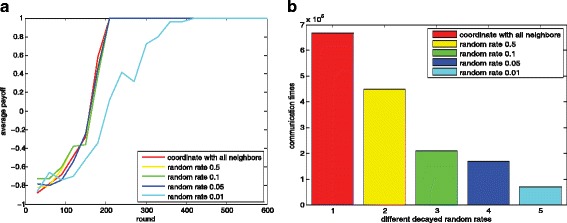
Selecting the coordination sets with different randomness. Figure 11**a** dynamics of the average payoffs using 4 different random parameters; Fig. 11**b** corresponding communication times

**Fig. 12:**
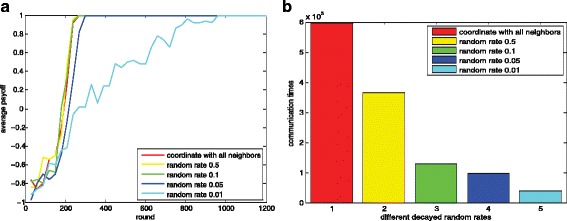
Selecting the coordination sets with different decayed randomness. Figure 12**a** dynamics of the average payoffs using 5 different initialized random parameters; Fig. 12**b** corresponding communication times

In this subsection, we evaluate the influence of random parameter *δ* introduced in “Coordination set selection: random” section. The parameter settings are shown in Table 2. Figure 11 show the learning process of the agents using different random coordination set selection methods. In Fig. 11a, we observe that all methods enable the agents to reach a global optimal policy with an average reward of 1. With the decrease of the random rate, more rounds are needed to reach a global optimal. And from Fig. 11b, we can see the corresponding communication times over the whole network are reduced. Figure 12 show the learning process of the agents using decayed random methods. The 4 methods are initialized with different *δ*_0_ and different decay rate (see Table 2 for detail). Figure 12a shows that with the decay of the initialization of *δ*_0_, more rounds are needed. And when decay is added to the random methods, the corresponding communication times are significantly decreased without infecting the convergence performance as shown in Fig. 12b. But when the initialized *δ*_0_ is too small (*δ*_0_=0.001), the added decay makes little difference to the communication but leads to more learning rounds.

**Fig. 13:**
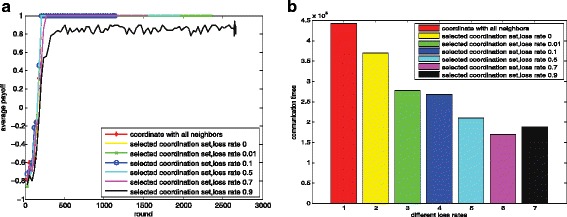
Selecting the coordination sets with different loss rate *δ*. Figure 13**a** dynamics of the average payoffs using different loss rates; Fig. 13**b** corresponding message passing times

**Table 2:** Parameter settings for “The influence of random parameter *δ*” section

Parameter name	Value
Agent number	100
Action number	10
Init explore rate	1.0
Delta explore rate	0.003
Init learning rate	1.0
Delta learning rate	0.0005
Min learning rate	0.6
Init random rate	1, 0.5, 0.1, 0.05, 0.01
Delta random rate	0.005, 0.002, 0.0004, 0.0002, 0.000004
Message differ(max-plus)	0.00001
Message sent deadline(max-plus)	5

#### The influence of loss rate *δ*

In this subsection, we investigate the influence of the loss rate *δ* defined in “Coordination set selection: loss rate” section. The parameter settings are shown in Table 3. We use the loss rate to identify the coordination set for each agent. The size of the coordination set decreases with the increase of the loss rate *δ*. The norm emergence performance with different loss rate *δ* and the corresponding communication times are shown in Fig. 13. From Fig. 13a, we see that the norm emergence efficiency is reduced as the increase of the loss rate *δ*. Given the other parameters unchanged, we see that when *δ*<=0.7, our method can significantly reduce the communication without influencing the learning performance. When *δ*>0.7, more time is needed for the agent to reach a global optimal. When *δ*>0.9, our method may fail to converge in a few steps with the same parameters and more exploration is needed. The corresponding message passing times over all agents are shown in Fig. 13b.

**Fig. 14:**
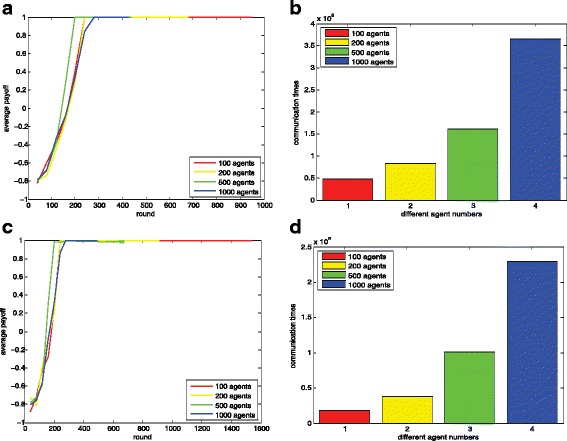
Influence of population size. Figure 14**a** dynamics of the average payoffs(random); Fig. 14**b** corresponding message passing times(random); Fig. 14**c** dynamics of the average payoffs(loss rate); Fig. 14**d** corresponding message passing times (loss rate);

**Table 3:** Parameter settings for “The influence of loss rate *δ*” section

Parameter name	Value
Agent number	100
Action number	10
Init explore rate	1.0
Delta explore rate	0.004
Init learning rate	1.0
Delta learning rate	0.0005
Min learning rate	0.6
Loss rate	None, 0, 0.01, 0.1, 0.5, 0.7, 0.9
Message differ(max-plus)	0.00001
Message sent deadline(max-plus)	5

#### The influence of population size *n*

The influence of the population size is shown in Fig. 14. We evaluate our methods in a group of agents range from 100 to 1000. The parameter settings are shown in Table 4. We can clearly observe the norm emergence efficiency is not influenced obviously as the increase of the population size. Through the passing of messages, the agents coordinate their actions in a few steps. And the results show that our method scales well in large systems. Figure 14a and b show the results using random methods and Fig. 14c and d show the results using loss rate controlled methods. The random rate and the loss rate here are set to 0.5. And we can clearly observe the message passing times over all agents are proportional to the number of agents from Fig. 14b and d.

**Table 4:** Parameter settings for “The influence of population size *n*” section

Parameter name	Value
Agent number	100,200,500,1000
Action number	10
Init explore rate	1.0
Delta explore rate	0.004
Init learning rate	1.0
Delta learning rate	0.0005
Min learning rate	0.6
Message differ(max-plus)	0.00001
Message sent deadline(max-plus)	5

## Conclusion

In this paper, we develop a framework based on the max-plus algorithm to accelerate the norm emergence of large cooperative MASs. With the limited communication bandwidth, we propose two kinds of approaches to minimize the communication cost: random and deterministic. Random methods select the coordination set stochastically, while the deterministic methods identify the best coordination set for each agent by limiting the utility loss due to the lock of coordination. Both approaches significantly reduce links of the coordination graph and result in less communication without deteriorating the learning performance. Experiment results show that our methods lead to better norm emergence performance under all kinds of networks compared with the existing methods and scale well in large populations. Thus, our methods can efficiently accelerate the social norm emergence under limited communication.

As future work, we will further investigate the performance of our methods in more complicated games such as Prisoner’s dilemma, to better reflecting the interaction dynamics in cell systems. And we will evaluate our algorithm on a simulated cell communication environment.
